# Chondroprotective effects of CDK4/6 inhibition via enhanced ubiquitin-dependent degradation of JUN in synovial fibroblasts

**DOI:** 10.1093/rheumatology/keab874

**Published:** 2021-11-25

**Authors:** Tadashi Hosoya, Tetsuya Saito, Hiroyuki Baba, Nao Tanaka, Seiji Noda, Youji Komiya, Yasuhiro Tagawa, Akio Yamamoto, Fumitaka Mizoguchi, Kimito Kawahata, Nobuyuki Miyasaka, Hitoshi Kohsaka, Shinsuke Yasuda

**Affiliations:** Department of Rheumatology, Graduate School of Medical and Dental Sciences, Tokyo Medical and Dental University (TMDU), Tokyo, Japan; Department of Rheumatology, Graduate School of Medical and Dental Sciences, Tokyo Medical and Dental University (TMDU), Tokyo, Japan; Department of Rheumatology, Graduate School of Medical and Dental Sciences, Tokyo Medical and Dental University (TMDU), Tokyo, Japan; Department of Rheumatology, Graduate School of Medical and Dental Sciences, Tokyo Medical and Dental University (TMDU), Tokyo, Japan; Department of Rheumatology, Graduate School of Medical and Dental Sciences, Tokyo Medical and Dental University (TMDU), Tokyo, Japan; Department of Rheumatology, Graduate School of Medical and Dental Sciences, Tokyo Medical and Dental University (TMDU), Tokyo, Japan; Department of Rheumatology, Graduate School of Medical and Dental Sciences, Tokyo Medical and Dental University (TMDU), Tokyo, Japan; Department of Rheumatology, Graduate School of Medical and Dental Sciences, Tokyo Medical and Dental University (TMDU), Tokyo, Japan; Department of Rheumatology, Graduate School of Medical and Dental Sciences, Tokyo Medical and Dental University (TMDU), Tokyo, Japan; Department of Rheumatology, Graduate School of Medical and Dental Sciences, Tokyo Medical and Dental University (TMDU), Tokyo, Japan; Department of Rheumatology, Graduate School of Medical and Dental Sciences, Tokyo Medical and Dental University (TMDU), Tokyo, Japan; Department of Rheumatology, Graduate School of Medical and Dental Sciences, Tokyo Medical and Dental University (TMDU), Tokyo, Japan; Department of Rheumatology, Graduate School of Medical and Dental Sciences, Tokyo Medical and Dental University (TMDU), Tokyo, Japan

**Keywords:** fibroblasts, rheumatoid arthritis, ubiquitin, AP-1, RNA-seq

## Abstract

**Objective:**

Targeting synovial fibroblasts (SF) using a cyclin-dependent kinase (CDK) 4/6 inhibitor (CDKI) could be a potent therapy for RA via inhibition of proliferation and MMP-3 production. This study was designed to elucidate the mechanism of chondroprotective effects on SFs by CDK 4/6 inhibition.

**Methods:**

CDK4/6 activity was inhibited using CDKI treatment or enhanced by adenoviral gene transduction. Chondroprotective effects were evaluated using a collagen-induced arthritis model (CIA). Gene and protein expression were evaluated with quantitative PCR, ELISA and Western blotting. The binding of nuclear extracts to DNA was assessed with an electrophoresis mobility shift assay. RNA-Seq was performed to identify gene sets affected by CDKI treatment.

**Results:**

CDKI attenuated cartilage destruction and MMP-3 production in CIA. In RASFs, CDKI impaired the binding of AP-1 components to DNA and inhibited the production of MMP-1 and MMP-3, which contain the AP-1 binding sequence in their promoter. CDK4/6 protected JUN from proteasome-dependent degradation by inhibiting ubiquitination. The RNA-Seq analysis identified CDKI-sensitive inflammatory genes, which were associated with the pathway of RA-associated genes, cytokine-cytokine receptor interaction and IL-17 signalling. Notably, the AP-1 motif was enriched in these genes.

**Conclusion:**

The mechanism of chondroprotective effects by CDK4/6 inhibition was achieved by the attenuation of AP-1 transcriptional activity via the impaired stability of JUN. Because the pharmacologic inhibition of CDK4/6 has been established as tolerable in cancer treatment, it could also be beneficial in patients with RA due to its chondroprotective and anti-inflammatory effects.

Rheumatology key messages

- CDK4/6 inhibition exerts chondroprotective effects in addition to inhibiting synovial fibroblast proliferation.
- CDK4/6 protect JUN from ubiquitin-dependent degradation, which was facilitated by CDK4/6 inhibitor.
- CDK4/6 inhibition would be beneficial for patients with rheumatoid arthritis when combined with other treatment modalities.

## Introduction

RA is one of the most common chronic inflammatory disorders [1]. Prolonged inflammation of the affected joints disturbs joint function and results in cartilage and bone destruction. Current inflammation-targeting therapy consists of biological and targeted synthetic disease-modifying anti-rheumatic drugs (DMARDs) that improve the outcomes of patients with RA [2]. However, achieving complete disease remission is still challenging [3] and joint function can still deteriorate even in patients who do reach clinical remission [4]. One explanation is that cartilage damage occurs in the early stages of RA and results in secondary joint dysfunction [5, 6]. Future treatments for RA should aim at preventing cartilage destruction in addition to ameliorating inflammation.

Synovial fibroblast (SF)-targeted therapy is a possible alternative or complementary therapeutic strategy that could be used to solve these problems. RA synovial fibroblasts (RASFs) are the main source of MMPs, which play a central role in the degradation of the cartilage matrix composed of aggrecan and collagen [7]. RASFs are epigenetically altered to enhance invasiveness as well as excessive cytokine-production and proliferating capacity in the inflamed joints of RA patients [8]. The genetic risk of RA was demonstrated by a trans-ethnic genome-wide association study [9]. Recently, significant overlap of the risk loci and regulatory regions were identified in multi-cytokine stimulated RASFs as well as in CD4 T cells and B cells [10, 11]. These findings indicate the pathologic contribution of SFs as local effector cells that results in a vicious circle of destruction in arthritic joints [12, 13].

Recently, seliciclib, an orally available cyclin-dependent kinase (CDK) inhibitor, was examined in patients with RA in early phase clinical trials [14, 15]. Additionally, genome-wide association studies identified multiple CDK family genes, *CDK4*, *CDK6* and *CDK2,* as RA risk genes [9, 16]. Because cell proliferation was strictly aligned with CDK activity, CDK inhibitors would be expected to direct or explicitly target their actions against SFs. We have demonstrated previously that the inhibition of CDK4/6 activity in RASF suppressed the production of inflammatory mediators, including MMP-3 [17]. Inhibition of CDK4/6 prevented joint destruction in animal models of arthritis by inhibiting synovial cell proliferation and perhaps by exerting chondroprotective effects in the arthritic joints [18, 19]. However, the underlining mechanism remains unclear, including the involved transcriptional factors.

In the present study, we observed chondroprotective effects in an animal model of arthritis and selective suppression of MMP-1/MMP-3 in RASFs using a CDK4/6 inhibitor. Under the inhibition of CDK4/6, the auto-amplification of JUN and FOSL1, components of AP-1, was impaired via enhanced ubiquitin dependent degradation of JUN protein. Furthermore, using unbiased transcriptomic analysis, we confirmed that the inhibition of CDK4/6 resulted in the selective suppression of genes that are regulated by JUN and FOSL1. These findings demonstrate a novel functional aspect of CDK4/6 regulation of inflammatory mediators and could provide an opportunity for the development of a complementary treatment for cartilage protection in RA.

## Materials and methods

### Reagents

The following antibodies were used: Anti-JUNB(ab128878), anti-JUND(ab134067), anti-FOSB(ab184938) and anti-FOSL2(ab124830) were purchased from Abcam (Cambridge, UK); anti-FOS(sc-8047), anti-FOSL1(#sc-183), anti-p-JNK(sc-6254) and anti-β-actin(AC-15) were purchased from Santa Cruz Biotechnology (Santa Cruz, CA, USA); anti-JUN(#9165), anti-JNK(#9252), anti–p-P38 MAPK(#9211), anti-P38(#9212), anti-p-ERK1/2(#9106), anti-ERK1/2(#9102), anti-ubiquitin(#3936) and anti-p-Rb(#9308) were purchased from Cell Signalling Technology (Danvers, MA, USA); anti-p-JUN (Ser73) (#06–659) was purchased from EMD Millipore (Temecula, CA, USA).

Palbociclib was synthesized by ChemieTek (Indianapolis, IN, USA). SP600125 and MG132 were purchased from Sigma-Aldrich (St Louis, MO, USA). All compounds were dissolved in dimethyl sulfoxide (DMSO). TNFα was purchased from Genzyme (Cambridge, MA, USA) and IL-1β was purchased from PeproTech (Rocky Hill, NJ, USA).

### Mice

Six-week-old male DBA/1J mice were purchased from the Japan Charles River Breeding Laboratories (Kanagawa, Japan) and maintained in the animal facility at the Tokyo Medical and Dental University. All procedures in the animal experiments were reviewed and approved by the Institutional Animal Care and Use Committee of the Tokyo Medical and Dental University (TMDU). Reference IDs were M2000-979 and G2018-028.

### Cartilage degeneration in collagen-induced arthritis (CIA)

The method of induction and evaluation was described previously [19]. Briefly, seven mice consisted of one group. Each mouse was immunized with 200 µg of bovine type collagen (Collagen Research Center, Tokyo, Japan) emulsified with complete Freund’s adjuvant (Difco Laboratories, Detroit, MI, USA) by injection at the tail base. Immunization was repeated 21 days after the primary immunization. CDKI or vehicle were dissolved in 0.5% methylcellulose and orally administrated to mice after the second immunization to the end of experiments. To evaluate cartilage destruction, we stained joint sections with toluidine blue. Cartilage degeneration was graded from 0 to 5 by the degree of cartilage loss and chondrocyte loss: 0, normal; 1, minimal loss of with no obvious chondrocyte loss; 2, mild loss of cartilage with superficial chondrocyte loss; 3, moderate loss of cartilage with chondrocyte loss above middle zone; 4, marked loss of cartilage with chondrocyte loss above deep zone; 5, severe diffuse loss of cartilage with chondrocyte loss to tidemark [20]. Cartilage depth was determined as the average of three areas.

### Cell culture

Synovial tissues were obtained from patients with RA when they underwent joint replacement surgery or synovectomy. Written informed consent was obtained before surgery. All the patients fulfilled the criteria of the ACR [21]. Synovial tissues were minced into small pieces and then subjected to enzymatic digestion using 2 mg/ml of collagenase type 4 (Worthington, NJ, USA), 0.8 mg/ml of Dispase II and 0.1 mg/ml of DNase I (Roche, Basel, Switzerland) in Dulbecco’s modified Eagle Roche’s medium (DMEM) at 37°C. After 15 min, we collected the supernatant and replaced with fresh enzyme mix. These procedures were repeated every 15 min for total 1 h. Isolated cells were cultured in DMEM supplemented with 10% FBS (Gemini Bio, CA, USA), antibiotics (penicillin and streptomycin), and passaged every week to avoid confluency. All experiments used proliferating RASFs (from passage 5–11). The study protocol was approved by the ethics committees of TMDU. Reference IDs were M2000-979 and G2018-028.

### PCR

RASFs were pre-treated overnight with 2 µM CDKI, or DMSO as a control, then stimulated with 0.2 ng/ml of IL-1β and TNFα for the indicated period. Messenger RNA levels of target genes in the treated RASFs were evaluated by quantitative PCR. RNA was extracted using a RNeasy Plus Mini Kit (QIAGEN, Tokyo, Japan). Complementary DNA was synthesized with QuantiTect Reverse Transcription kit (QIAGEN). Quantitative PCR was performed using a SYBR Green RT-PCR Kit (QIAGEN) with sets of primers specific for each gene.

### ELISA

To analyse serum levels of MMP-3 in CIA mice, blood samples were collected on day 42. The cytokine levels in the supernatant of RASFs were determined by ELISA. All kits were purchased from R&D Systems (Minneapolis, MN, USA).

### Electrophoresis mobility shift assay (EMSA) and DNA ELISA

RASFs were pre-treated overnight with 2 µM CDKI, 20 µM SP600125 (JNK inhibitor), or DMSO as a control, then stimulated with 0.2 ng/ml of IL-1β and TNFα for 6 h. Nuclear lysates were prepared with a Nuclear Extraction kit (Active Motif, Carlsbad, CA, USA). EMSA was performed using a second-generation gel shift assay kit (Roche, Tokyo, Japan) and DNA ELISA performed using TransAM AP-1 Transcription Factor ELISA Kits (Active Motif).

### Western blotting (WB)

RASFs were pre-treated with or without 2 µM CDKI and/or 1 µM MG132, overnight. Cells were then stimulated with 0.2 ng/ml of TNFα and IL-1β for the indicated period. Two% SDS buffer containing complete mini protease inhibitors (Roche, Tokyo, Japan) and a phosphatase inhibitor cocktail (Sigma-Aldrich, St Louis, MO, USA) was used for sample preparation. To reduce and denature the protein, cell lysates were boiled at 95 degrees for 5 min with 5% 2-mercaptoethanol. Then, samples were separated by gel electrophoresis and transferred onto polyvinylidene difluoride membranes. Membranes were blocked with 5% bovine serum albumin for 1 h at room temperature and were incubated with the optimally diluted first antibody overnight at 4 degrees. Peroxidase-conjugated IgG Ab (Cell Signalling Technology) was used as a secondary Ab. To quantify the signal intensity, we performed densinometric analyses using ImageJ software (National Institutes of Health, USA), shown in Supplementary Fig. S1, available at *Rheumatology* online.

### Immunoprecipitation

pLX304 vector carrying human-UBB gene was provided from the research core unit at TMDU. RASFs were transduced with lentivirus expressing ubiquitin B. After 5 days of blasticidin selection, transduced RASFs were pre-treated with a combination of 1 µM MG132 and/or 2 µM CDKI overnight and stimulated with 0.2 ng/ml of TNFα and IL-1β for 6 h. Then, samples were lysed with RIPA lysis buffer (EMD Millipore) containing cOmplete mini protease inhibitor^TM^ (Sigma Aldrich, St Louis, MO, USA), 10 µM iodoacetamide, and 10 µM N-ethylmaleimide. JUN antibody was immobilized on FG beads (Tamagawa Seiki Co. Ltd, Nagano, Japan) according to the manufacturer’s protocol. The protein solution was incubated with the antibody conjugating beads on a rotator for 2 h at 4 degrees. After the incubation, immunoprecipitates were eluted with an acid solution after the beads were separated magnetically. The immunoblotting was done according to the WB protocol.

### RNA-Seq data processing

We prepared five RASF samples from each donor under four conditions: in the presence or absence of CDKI and with or without cytokine stimulations. In total, 20 samples were analysed. RNAs were obtained using a RNeasy Plus Mini Kit (QIAGEN) for the library. Sequencing was performed on DNBSEQ platform with 150-bp paired-end reads (MGISEQ-2000RS) to a depth of 50–60M reads per library. Reads containing low quality bases, adaptor contamination, or excessively high levels of unknown base N were removed. Filtered reads were aligned for the GRCh38 (patch release 12) by Bowtie2 (v2.2.5; http://bowtie-bio.sourceforge.net/bowtie2/index.shtml). Mapping rates were >90% in all samples. Gene expression was quantified using RSEM [22]. In total, 17 873 genes were expressed (RPKM > 0) in more than one sample and further analysed. The expression data were normalized for heatmap between samples with a trimmed mean of M values [23]. The values for each gene were inverse normal transformed across samples.

### Differentially expressed genes (DEGs)

Genes expressing significantly different amounts between the paired groups were determined using the PossionDis method based on poisson distribution [24] and DESeq2. FDR ≤ 0.001 in PossionDis and Qvalue (adjusted *P*-value) ≤0.05 in DESeq2 were considered as cutoffs.

### Enrichment analysis

The phyper function in R software was used to evaluate the enrichment of gene sets. Qvalue ≤0.05 is regarded as a significant enrichment. For the pathway classification, KEGG annotation was used. For transcription factor (TF) enrichment analysis, we used ChIP-atlas [25]/ChEA3 data [26].

### Statistical analysis

Means (s.e.m.) were shown in the analysis. Student’s *t test* was used to compare the two conditions. Dunnett’s test was used to compare more than three conditions of the treated samples against vehicle treated or control siRNA-treated samples. *P* < 0.05 were considered statistically significant. Prism 5 (GraphPad Software, San Diego, CA, USA) statistical software was used to carry out the analysis.

## Results

### CDK4/6 inhibitor demonstrated chondroprotective effects in collagen-induced arthritis and suppressive effects on MMP-1 and MMP-3 production from RASFs

**Fig. 1:**
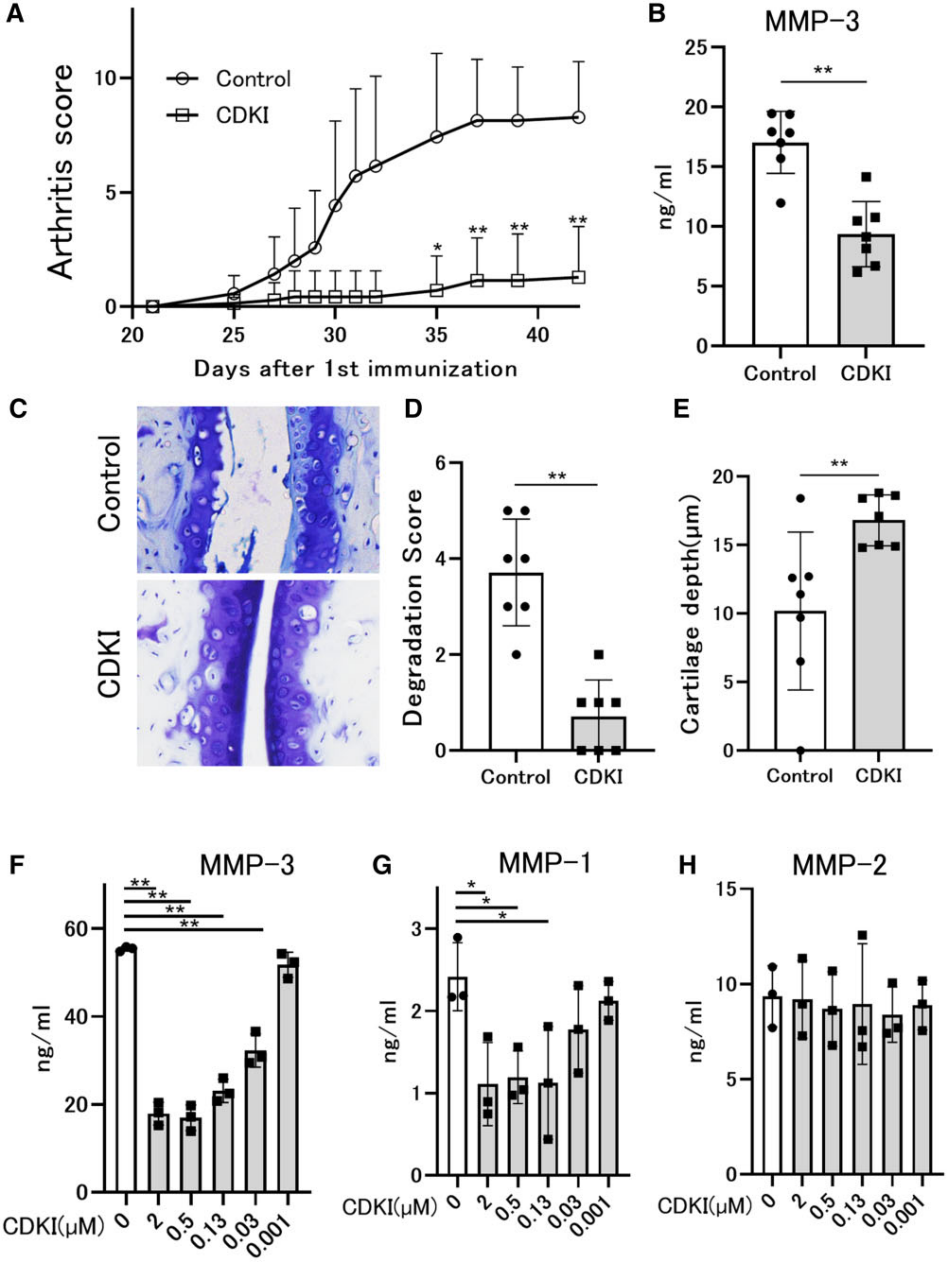
Chondroprotective effects in collagen-induced arthritis and MMP-1 and MMP-3 suppression in RASFs by CDKI (**A**) CIA mice (*n* = 7) were treated with oral administration of 100mg/kg of CDKI or vehicle from 21 days until 30 days after the initial immunization. Severity of arthritis was assessed as arthritis scores. Data represent the mean and SE. The statistical significance of differences between control group and treated group was determined (*P < 0.05; **P < 0.01). (**B**) Serum level of MMP-3 on day 42. (**C**–**E**) Joint samples were collected on day 42, stained with toluidine blue (**C**), and analysed histologically for cartilage degeneration score (**D**) and cartilage depth (**E**). Data were analysed by Student’s t-test. (*P < 0.05; **P < 0.01). Data were representative of two independent experiments showing similar results. (**F**–**H**) The cytokine-induced production of MMP-1, MMP-2 and MMP-3 in RASFs treated with indicated concentration of CDKI. RASFs were pre-treated with or without CDKI overnight, then stimulated with the combination of TNFa and IL-1b at 0.2 ng/for 72 hours. Data were analyzed by Dunnett’s test. (*P < 0.05; **P < 0.01). Data were representative of three independent experiments showing similar results.

As we have shown previously [19], CDK4/6 selective inhibitor (CDKI), palbociclib suppressed the severity of arthritis (Fig. 1A). In the CDKI-treated mice, the serum level of MMP-3 was suppressed (Fig. 1B) and cartilage degradation was prevented in the tibia-talus joint (Fig. 1C and E).

CDKI suppressed MMP-1 similar to MMP-3, but MMP-2 was not suppressed in RASFs (Fig. 1F–H).

### Nuclear AP-1 family proteins were decreased by CDKI treatment

**Fig. 2:**
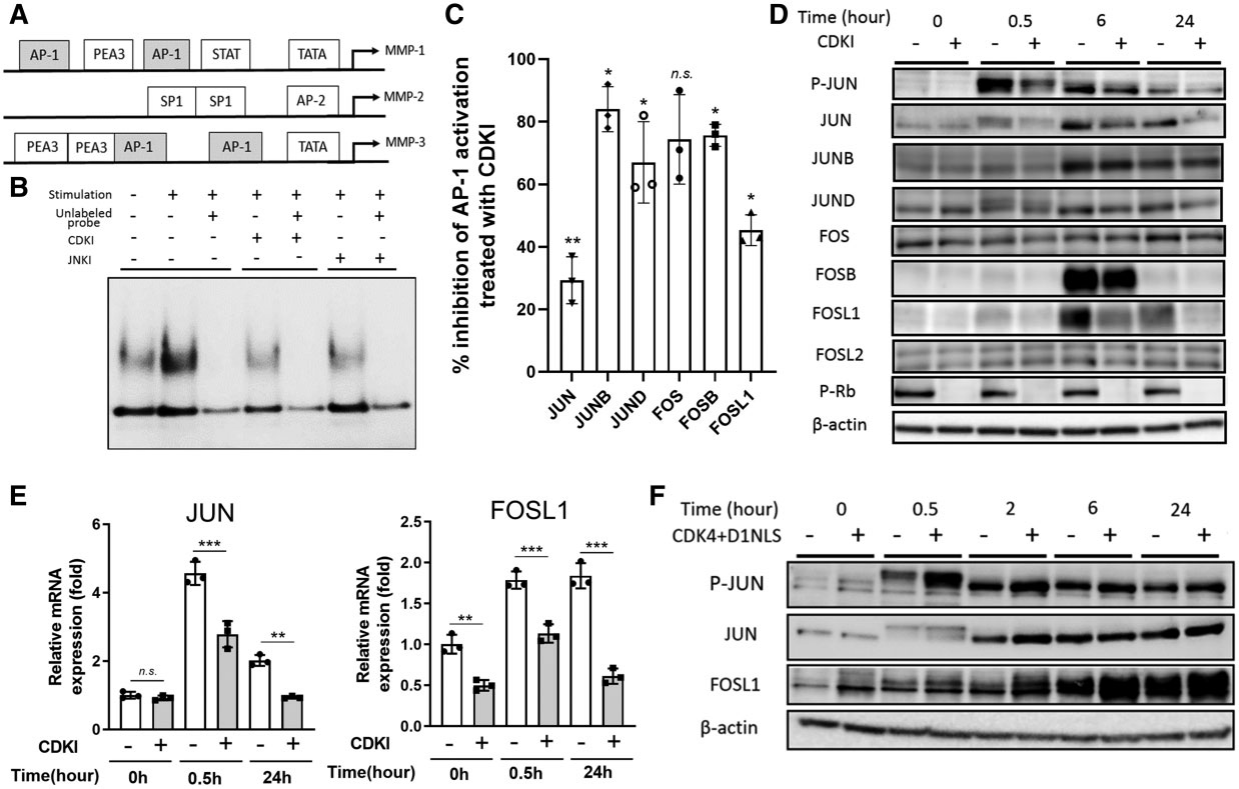
CDK4/6 activity regulated the expression and induction of JUN and FOSL1 (**A**) Transcription factor binding sites in the promoter of MMP-1, MMP-2 and MMP-3 genes. The transcription start sites are indicated with the bent arrow. Transcription factor-binding sites include the activator proteins (AP)-1 and -2 site, the nuclear factor of κB (NF-κB) site, the polyomavirus enhancer-A binding-protein-3 (PEA3) site, the stimulatory protein-1 site (SP1), the signal transducer and activator of transcription (STAT) site and the TATA-box (TATA). (**B**, **C**) The binding activity of the nuclear proteins to the AP-1 consensus sequence examined with EMSA and DNA-ELISA. The inhibitory effects were compared with the vehicle treated sample as 100%. Regarding each AP-1 component, data were analysed by Student’s t-test for comparing CDKI-treated against vehicle treated, respectively. (*P<0.05; **P<0.01). (**D**) The expression of AP-1 components (JUN, JUNB, JUND, FOS, FOSB, FOSL1 and FOSL2) and the phosphorylation of JUN at Ser 73 with or without CDKI at different time points after the cytokine stimulation. Retinoblastoma protein (Rb) is a substrate of CDK4/6. β-actin was used as a loading control. (**E**) The decreased transcription of JUN and FOSL1 by CDKI. The mRNA results were normalized to that of 18s mRNA and presented as fold change relative to non-treated cells. Data were analysed by Student’s t-test for comparing CDKI-treated samples against vehicle treated, respectively. (*P <0.05; **P <0.01). (**F**) The expression and induction of JUN and FOSL1 after the cytokine stimulation in RASFs with enhanced CDK4 kinase activity. RASFs were transduced with adenovirus expressing CDK4 and cyclin D1(CCND1)-nuclear localization signal, or lacZ as a control, and were stimulated with the cytokines for the indicated period. The expression and phosphorylation of the proteins were evaluated by WB. Data were representative of two independent experiments showing similar results.

Comparing the promoter structures, *MMP-1* and *MMP-3,* but not *MMP-2,* contained the phorbol 12-O-tetradecanoate-13-acetate (TPA) response element (TRE), which is an AP-1 binding sequence, upstream to the transcription starting site (Fig. 2A).

The amount of nuclear protein binding to TRE increased after cytokine stimulation and decreased with JNKI or CDKI pre-treatment (Fig. 2B). AP-1 forms a dimeric complex of two components that are structurally and functionally related members of the JUN and FOS families [27]. Among the AP-1 components, JUN, JUNB, JUND, FOSB and FOSL1 were decreased in the nuclear extract after CDKI treatment compared with the control (Fig. 2C). These findings suggest that CDKI inhibits MMP-1 and MMP-3 production in an AP-1 dependent manner.

### The cytokine-induced expression of JUN and FOSL1 was associated with the CDK4/6 activity

In CDKI-treated RASFs, the phosphorylation of JUN and the expression of JUN and FOSL1 were attenuated (Fig. 2D), indicating that CDKI downregulated JUN and FOSL1. In contrast, the expression of the other AP-1 components JUNB, JUND, FOS, FOSB and FOSL2 was not affected by CDKI treatment. We examined the expression of JUN and FOSL1 mRNA by quantitative PCR. CDKI inhibited JUN and FOSL1 after cytokine stimulation as well as the basal FOSL1 expression (Fig. 2E).

To exclude the possibility of off-target effects, we enhanced CDK4/6 activity directly by adenoviral transduction. RASFs were co-transduced with *CDK4* and *CCND1*(CDK4/D1) genes. Consistent with the previous results [17], the CDK4/D1-transduced RASFs showed increased DNA synthesis and enhanced productions of MMP-1 and MMP-3, but not MMP-2 (Supplementary Fig. S2A–D, available at *Rheumatology* online). The phosphorylation of JUN and the expression of JUN and FOSL1 were enhanced in CDK4/D1-transduced RASFs (Fig. 2F).

### JUN played an essential role in MMP-3 production and FOSL1 expression

**Fig. 3:**
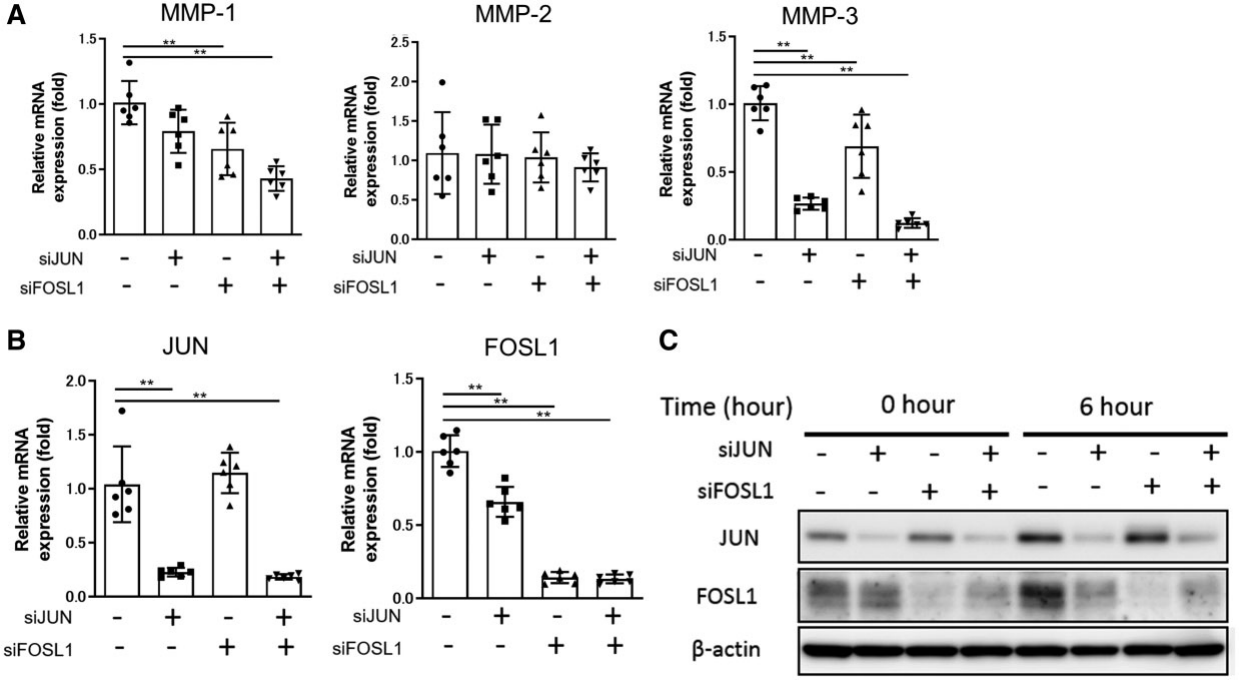
The essential contribution of JUN and FOSL1 to MMP production (**A–B**) The expression of MMP-1, MMP-2, MMP-3, JUN and FOSL1 mRNAs in the small interfering RNA (siRNA)-treated RASFs. JUN and FOSL1 were depleted by siRNA in RASFs. After 48 hours, siRNA-treated RASFs were stimulated with 0.2 ng/ml of TNFα and IL-1β for 6 hours. The results of mRNA were normalized to those of 18s and presented as fold change relative to scramble siRNA-treated cells. Data were analysed by Dunnett’s test. (*P <0.05; **P <0.01). (**C**) The expression of JUN and FOSL1 proteins in siRNA-treated RASFs. The protein expressions of JUN, FOSL1 and β-actin were evaluated by WB with or without 0.2 ng/ml of TNFα and IL-1β. Data were representative of two independent experiments showing similar results.

To explore the essential factor in the suppression of MMP-1 and MMP-3 by CDKI, we depleted JUN, FOSL1 or both genes using RNA interference. The induction of MMP-3 was inhibited by the knockdown of either JUN or FOSL1 or the combination. In turn, the induction of MMP-1 was inhibited by the knockdown of FOSL1 or the combination but not by the single knockdown of JUN (Fig. 3A). No inhibition was observed in MMP-2.

Because the transcriptions of JUN and FOSL1 genes are regulated by AP-1 binding to their promoters [28, 29], their transcriptional regulation is an auto-amplification. Additionally, the transcription and expression of FOSL1 induced by cytokine stimulation was inhibited in JUN small interfering RNA (si)- treated RASFs, indicating the regulation of FOSL1 by JUN (Fig. 3B and C). These findings indicated that MMP-3 was a direct-target of JUN, while MMP-1 was suppressed with CDKI via the down-regulation of FOSL1.

### CDK4/6 activity stabilized JUN protein by inhibiting ubiquitination and proteasome-dependent degradation

TNFα and IL-1β activated MAPKs, namely ERK1/2, JNK1/2 and P38, which are upstream of JUN and FOSL1. However, the phosphorylation status of these three MAPKs was not affected by CDKI (Supplementary Fig. S3A, available at *Rheumatology* online).

Because the down-regulation of JUN and FOSL1 by CDKI depended on the duration of pre-incubation (Supplementary Fig. S3B, available at *Rheumatology* online), we considered that the degradation of JUN was enhanced with inactive CDK4/6 and resulted in the attenuated cytokine responses.

**Fig. 4:**
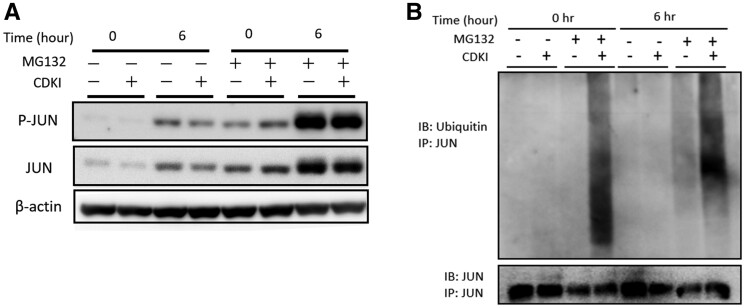
The downregulation and impaired induction of JUN by CDK4/6 inhibition were mediated by enhanced ubiquitination and proteasome-dependent degradation (**A**) The phosphorylation at Ser 73 and the expression of JUN with the combination treatment of CDKI and/or MG132, a proteasome inhibitor. The indicated proteins were analyzed by WB. (**B**) The enhanced ubiquitination on the JUN protein by CDKI. RASFs were transduced with lentivirus expressing ubiquitin B. Immunoprecipitates were eluted from JUN antibody conjugating beads and were analysed by immunoblotting using anti-ubiquitin or anti-JUN antibodies. Data were representative of two independent experiments showing similar results.

The mechanism of JUN degradation was reported to be proteasome-dependent [27]. Thus, we analysed the co-incubation of CDKI with MG132, a proteasome inhibitor. After treatment with MG132, the expression level of JUN was increased (Fig. 4A). The combination treatment of CDKI with MG132 abrogated the down-regulation of JUN expression level, indicating that CDK4/6 protected JUN from proteasome-dependent degradation (Fig. 4A).

To find out whether the ubiquitination status of JUN was affected by CDKI, we performed an immunoprecipitation analysis of JUN. The anti-JUN immunoprecipitates derived from ubiquitin B transduced RASFs treated with CDKI and MG132 were analysed by immunoblot of ubiquitin and JUN. In the presence of MG 132, the ubiquitinated JUN was increased in the CDKI-treated RASFs regardless of the cytokine stimulation (Fig. 4B).

### CDK4/6 inhibition repressed RA-associated genes as well as cell cycle associated genes

We hypothesized that the enhanced degradation of JUN was the central mechanism of the anti-inflammatory effects of CDKI. To assess the hypothesis, we performed RNA-seq for analysing the global changes of gene expression by CDK4/6 inhibition in RASFs.

**Fig. 5:**
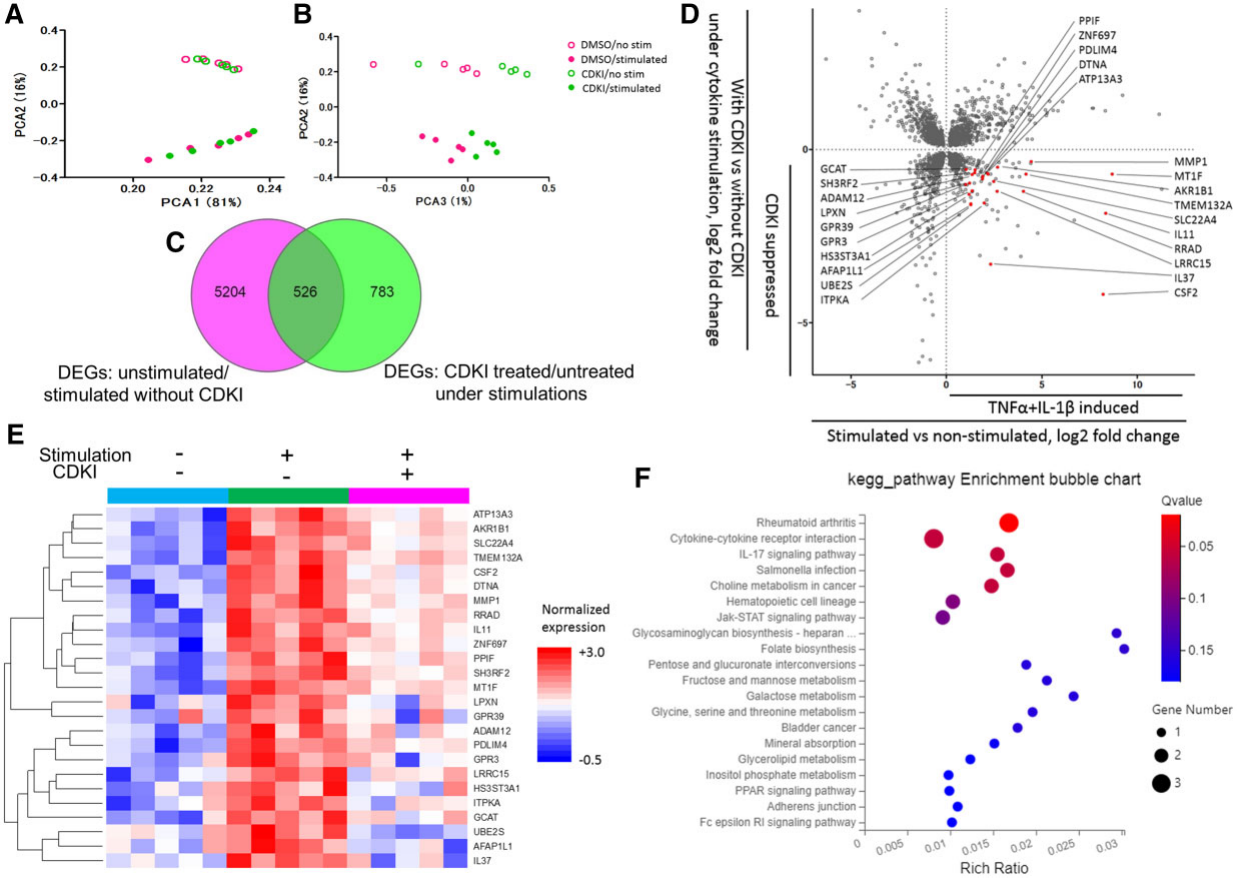
The transcriptomic features affected by CDK4/6 inhibition in cytokine stimulated RASFs (**A**, **B**) Principal component (PC) plot of gene expression data (17 873 genes). Proportion of variance derived from PC1, PC2 and PC3 occupied 81%, 16% and 1%, respectively. Each dot represents an independent sample. (**C**) Venn diagram of differentially expressed genes (DEG) identified in two analyses. The areas superimposed by two circles represent the intersection of these gene sets. The numbers on the figure indicate the number of genes in the corresponding area. (**D**) Scatter plot indicating the fold change (FC) in DEG analyses. In total, 2298 genes (FDR adjusted *P*<0.1) were used for the plot. XY axes represent log2 fold change. We labelled 25 genes in red, which fulfilled the additional criteria: induced by cytokine stimulation (Log2 fold above 1) and suppressed by CDK4/6 inhibition (Log2 fold below 0). (**E**) Heatmap of 25 genes in the non-stimulated (sky-blue), stimulated without CDKI (green), or stimulated with CDKI (magenta) samples. Each column indicates a sample with a colour demonstrating each gene expression level. Red colour indicates higher expression levels, blue colour indicates lower expression levels. (**F**) Pathway enrichment analysis of 25 genes. Colour in each plot indicates the value of FDR. The number of genes included in each pathway are represented by size of plot.

The major principal components exhibited differences under four conditions, which pointed out the transcriptome profiles affected by cytokine stimulation and CDK4/6 inhibition (Fig. 5A and B). PCA1/2 and PCA2/3 plots reflect changes in gene expression affected by the cytokine stimulation and CDK4/6 inhibition, respectively.

Differentially expressed genes (DEGs) were assessed in two ways: by comparing the unstimulated group and stimulated group without CDKI, and by comparing the CDKI-treated group and untreated group after cytokine stimulation. We identified 526 genes as DEGs by the two analyses (Fig. 5C). We focussed on the genes that were induced by the cytokine stimulation and inhibited by CDKI. Among the 526 genes, 25 were selected using the following criteria; induced by cytokine stimulation [Log2 fold change (FC) above 1] and inhibited by CDKI (Log2 FC below 0) (Fig. 5D). These 25 genes, named as CDKI-sensitive inflammatory genes, responded similarly among all individuals (Fig. 5E).

According to the KEGG pathway annotation, CDKI-sensitive inflammatory genes were enriched in RA-associated genes (Q value 0.03), cytokine-cytokine receptor interaction pathway (Q value 0.07) and IL-17 signalling pathway (Q value 0.07) (Fig. 5F).

To confirm that CDK4/6 inhibition resulted in cell cycle arrest at G1/S transition, we analysed DEGs between the CDKI-treated group and untreated group without stimulation. In total, 470 genes were suppressed by inhibition of CDK4/6 (Supplementary Fig. S4A, available at *Rheumatology* online) and were enriched in cell cycle or DNA replication pathway as expected (Supplementary Fig. S4B, available at *Rheumatology* online).

### JUN and FOSL1 were enriched in the CDKI-sensitive inflammatory genes

**Fig. 6:**
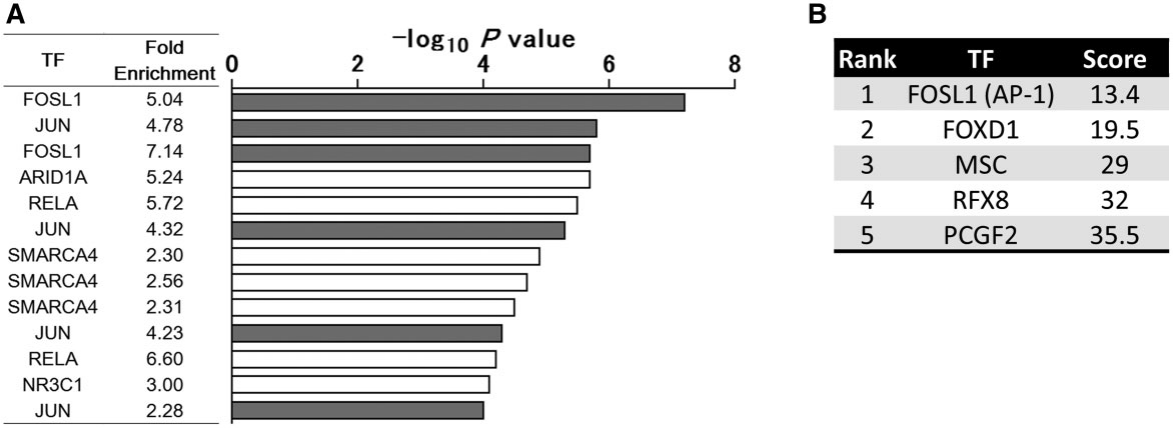
JUN and FOSL1 were enriched in the CDKI-sensitive inflammatory genes (**A**) The results of enrichment analysis using ChIP-atlas data. Transcription factors (TFs) enriched in CDKI-sensitive inflammatory genes are shown. (**B**) TFs enriched in CDKI-sensitive inflammatory genes were ranked using ChEA3 analysis (https://maayanlab.cloud/chea3/).

To identify the specific TF responsible for the regulation in CDKI-sensitive inflammatory genes, we performed TF enrichment analysis using ChIP-Atlas data. Among the several hundred candidates, JUN and FOSL1 were enriched around the transcription starting site (TSS) of CDKI-sensitive inflammatory genes, which was validated by multiple ChIP-data (Fig. 6A). Furthermore, we obtained comparable results using another platform, ChEA3. It also revealed that FOSL1 was the most relevant transcription factor (Fig. 6B).

## Discussion

Here, we confirmed the chondroprotective effects of CDKI in an animal model of arthritis. The selective suppression of MMP-1 and MMP-3 by CDKI was caused by attenuation of AP-1 transcriptional activity. Because JUN protein was protected from ubiquitin-proteasome dependent degradation when CDK4/6 were active, the inhibition of CDK4/6 resulted in the downregulation of the baseline JUN expression and its induction in RASFs. We hypothesized that the inhibition of CDK4/6 would suppress inflammatory mediators in the RASFs by enhancing JUN degradation and by impairing JUN and FOSL1 dependent transcriptional activity (Supplementary Fig. S5, available at *Rheumatology* online).

The RNA-seq analysis revealed that the inhibition of CDK4/6 resulted in suppression of selective genes in which JUN and FOSL1 motifs were enriched. These results were consistent with our findings and further supported the hypothesis of the regulatory mechanisms of MMP-1 and MMP-3 by CDKI.

Because cartilage destruction is the initial event resulting in joint destruction in RA, the prevention of ECM degradation would be a complementary treatment together with anti-inflammatory treatments. Although the chondroprotective effects of CDKI might result from attenuated inflammation, CDKI suppressed the tissue-degrading enzymes including MMP-1 and MMP-3. Additionally, CDKI might be beneficial to enhance chondrocyte maturation because CDK6 inhibited differentiation and maturation of chondrocyte [30, 31]. The development of MMP inhibitors for the treatment of patients with RA or OA failed because of the unfavourable musculoskeletal problems induced by non-specific inhibition [32]. Palbociclib has already been approved in patients with advanced breast cancer [33, 34]. Interestingly, the adverse events in musculoskeletal symptoms were similar or might be less frequent in patients treated with palbociclib [35]. Because the inhibition of CDK4/6 reduced the excess MMPs induced by cytokines from synovial fibroblasts, the combination use of CDKI with anti-inflammatory agents such as biologic agents might be an ideal therapeutic strategy as we have previously shown in an animal model of arthritis [19].

For targeting synovial fibroblast proliferation, CDK inhibitor would be a promising candidate. Early phase clinical trials using seliciclib, a multiple CDK inhibitor, combined with TNF inhibitor are ongoing [14, 15]. Recently, palbociclib treatment ameliorated the clinical symptoms and decreased serum levels of CRP and MMP-3 in patients with advanced breast cancer and RA [36]. Various CDK4/6 inhibitors currently under development for clinical applications have a favourable safety profile, indicating that they are potential candidates for drug repositioning to RA treatment [37]. Furthermore, a novel CDK4/6 inhibitor has been discovered for potential drug development for RA by Teijin Pharma [38].

Our results demonstrated that the pharmacological inhibition of CDK4/6 is a potent strategy for normalizing the inflammatory phenotype of pathogenic SFs. A recent study demonstrated that the AP-1 motif is enriched in specifically modified epigenetic regions in RASFs [8]. Similarly, AP-1 was the most enriched transcription factor in the TNF-induced genes with prolonged activation in RASFs [39]. These findings indicated that the inflammatory characteristic of RASFs was determined by the AP-1 activity, at least partially. Systemic AP-1 targeted therapy has been examined using T-5224 with favourable results in an animal model of arthritis and has proceeded to a phase II clinical trial for patients with RA [40]. However, no results have been reported after the trial ended in 2013. Because AP-1 plays a critical role in the development of and homeostasis in bone and neurons, complete inhibition would have safety concerns [41]. Compared with T-5224, the inhibitory effects of CDKI in cytokine-induced AP-1 activity was limited as shown above, suggesting its favourable properties.

We must acknowledge several limitations in this study. While the retinoblastoma (Rb) gene product is a well-known substrate of CDK4/6, NF-kB and FOXM1 were discovered as additional substrates [42, 43]. Although FOXM1 could mediate the protective effects on JUN protein [44], we failed to detect its expression as the expression level of FOXM1 was weaker in the non-tumour cells than in the tumour cells [45]. Additionally, we could not exclude the possibility that our observation could reflect the physiological differences between the proliferating and synchronized cells. It was reported that the expression of AP-1 components fluctuated during cell cycle progression [46, 47]. Because two kinds of E3 ubiquitin ligases, APC and SCF, are activated in turn in proliferating cells [48], JUN may be preferentially degraded in the synchronized RASFs at G1 phase by CDKI treatment. Several studies demonstrated the kinase-independent interaction of CDK6 with NF-kB, STAT3 and JUN [49, 50]. These findings suggest the possibility that CDK4/6 regulate inflammatory pathways other than the Rb-E2F pathway. In our study, inflammatory genes suppressed under the treatment of CDKI were focussed as CDKI-targeted genes. It might underestimate the cartilage protecting factors enhanced by CDKI.

Our study demonstrates a novel mode of action of CDKI. The inhibition of CKD4/6 provided chondroprotective effects via downregulation of AP-1 as well as by the inhibition of pannus formation, and may normalize the inflammatory characteristics of synovial tissues, thereby overcoming the vicious circle of chronic inflammation in arthritic joints.

## Supplementary Material

keab874_Supplementary_Data

## Data Availability

The data that support the findings of this study are available from the corresponding author upon request. RNA-Seq data is available in ArrayExpress (accession no. E-MTAB-1079).
