# Supplementary material for: Chondroprotective effects of CDK4/6 inhibition via enhanced ubiquitin-dependent degradation of JUN in synovial fibroblasts
Source: Rheumatology (Oxford). 2021 Nov 25;61(8):3427–38. doi: 10.1093/rheumatology/keab874 (PMC9348617; doi:10.1093/rheumatology/keab874)
Supplement: keab874_Supplementary_Data [file keab874_supplementary_data.docx]

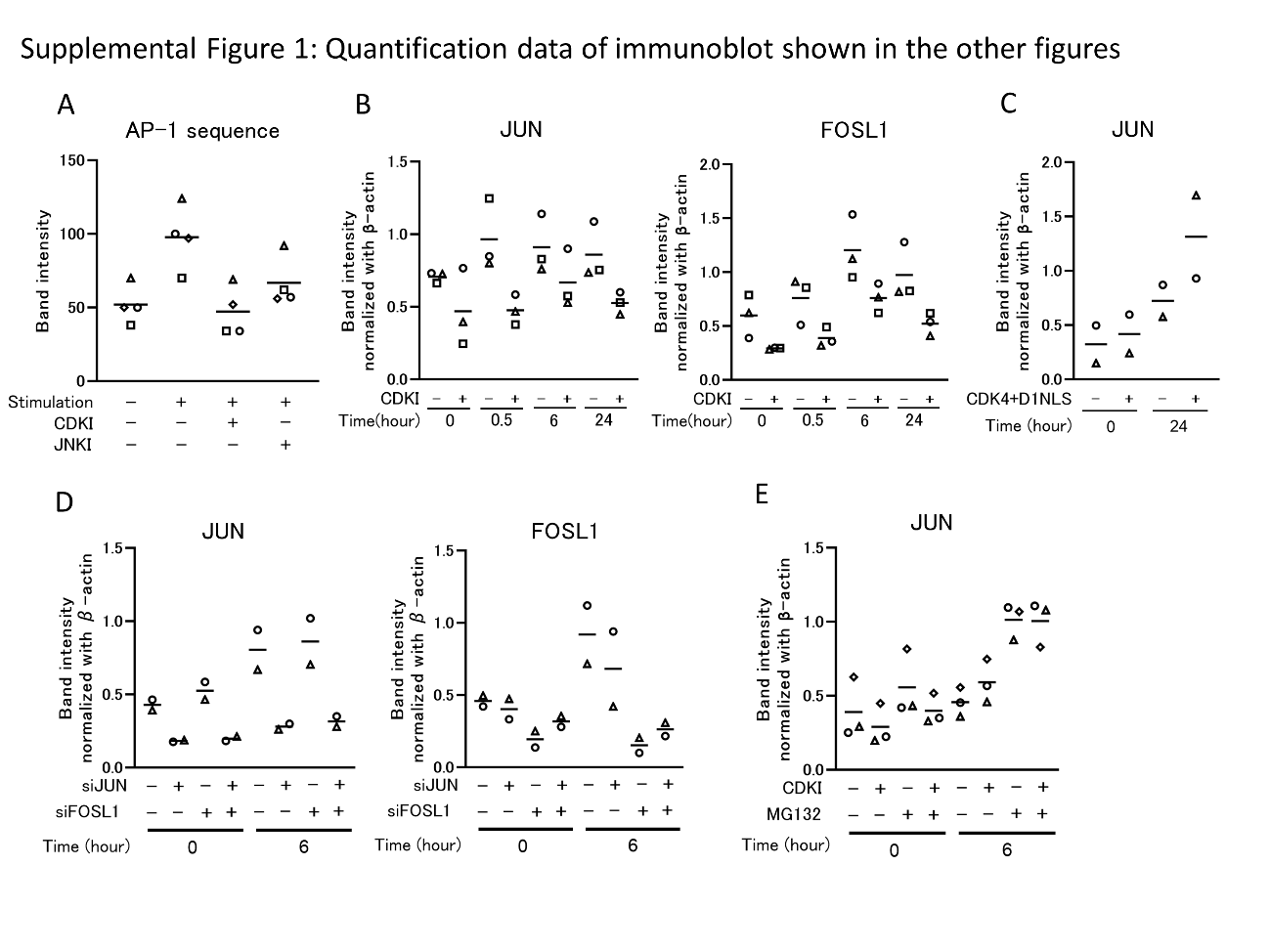


Supplementary Figure S1: Quantification data of immunoblot shown in the other figures.

The signal intensities of the immunoblot analysis were quantified using ImageJ and normalized with the internal control. We plotted the results from the repeated experiments to show the distribution using individual RASFs strains and the range of responses. Each symbol indicated the data derived from the single experiment.

A, D: Quantified data including repeated experiments of Figure 2B and 3C. B, C, E: Quantified data including repeated experiments of a part of Figure 2D, 2F and 4A, respectively.


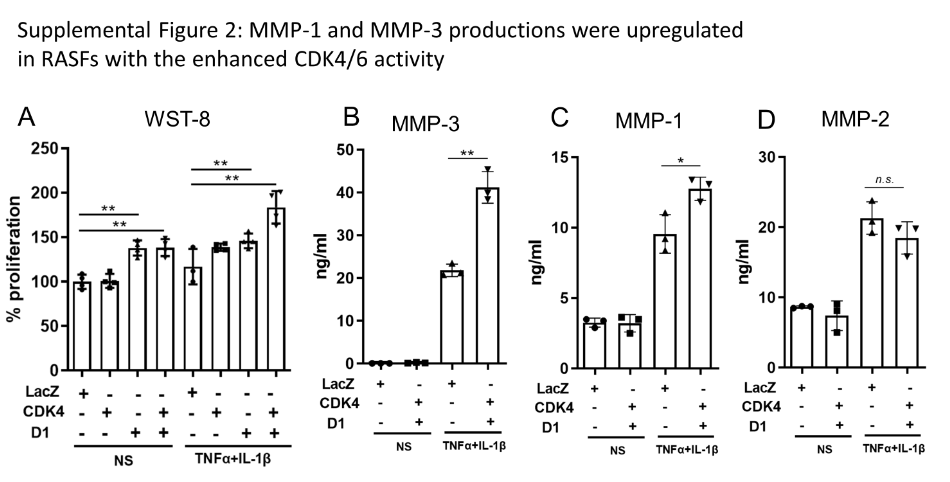


Supplementary Figure S2: MMP-1 and MMP-3 productions were upregulated in RASFs with the enhanced CDK4/6 activity.

A-D: RASFs were transduced with LacZ, CDK4, or cyclin D1-nuclear localization signal (D1) using adenovirus particle and were stimulated with 0.2ng/ml each of TNFα and IL-1β for 72 hours. Cell viability was evaluated with WST-8 kit (A). The levels of MMP-1, MMP-2, and MMP-3 in the supernatants were measured by specific ELISA kits (B-D). Data were analyzed by Dunnett’s test or Student’s t-test for comparing each sample against LacZ transduced. (*p<0.05, **p<0.01). Data were representative of two independent experiments showing similar results.


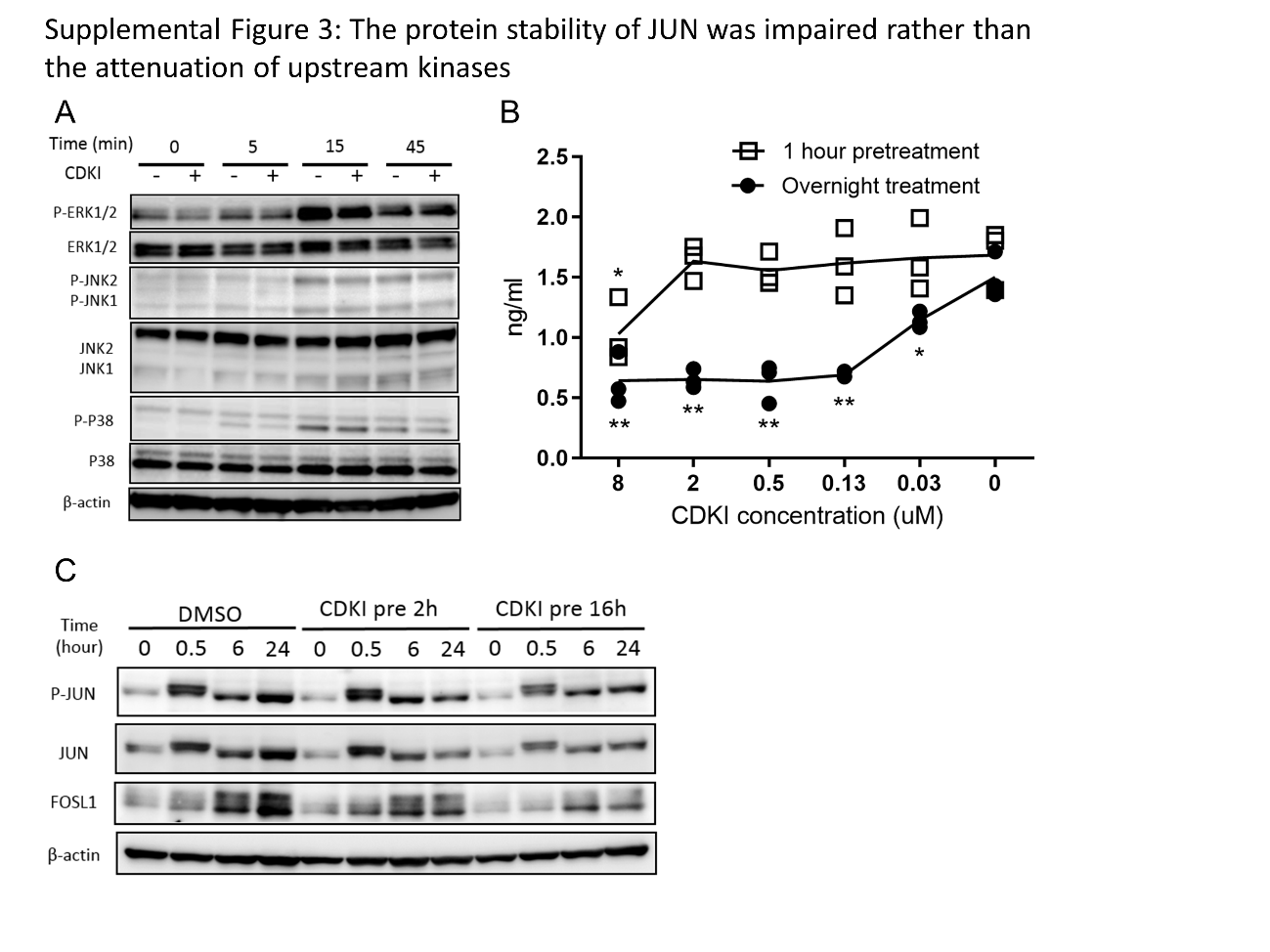


Supplementary Figure S3: The protein stability of JUN was impaired rather than the attenuation of upstream kinases

A: The phosphorylation of MAPKs (p-JNK, p-p38, and p-Erk1/2) at different time points with or without CDKI treatment. RASFs were pre-treated with 2µM CDKI overnight and stimulated with 0.2ng/ml each of TNFα and IL-1β for the indicated period. The phosphorylation and expression of MAPKs were analyzed by WB up to 45 minutes after the cytokine stimulation.

B: MMP-3 production suppressed by sustained but not temporal inhibition of CDK4/6. RASFs were treated with CDKI at the indicated concentrations overnight or for 1 hour, then stimulated with 0.2ng/ml of IL-1β and TNFα. Data were analyzed by Dunnett’s test. (*p<0.05, **p<0.01).

C: RASFs were pre-treated with 2µM CDKI for 2 hours or overnight and stimulated with 0.2ng/ml each of TNFα and IL-1β for the indicated period. The phosphorylation of JUN, and the expression of JUN, FOSL1 and β-actin were analyzed by WB up to 24 hours after the cytokine stimulation.


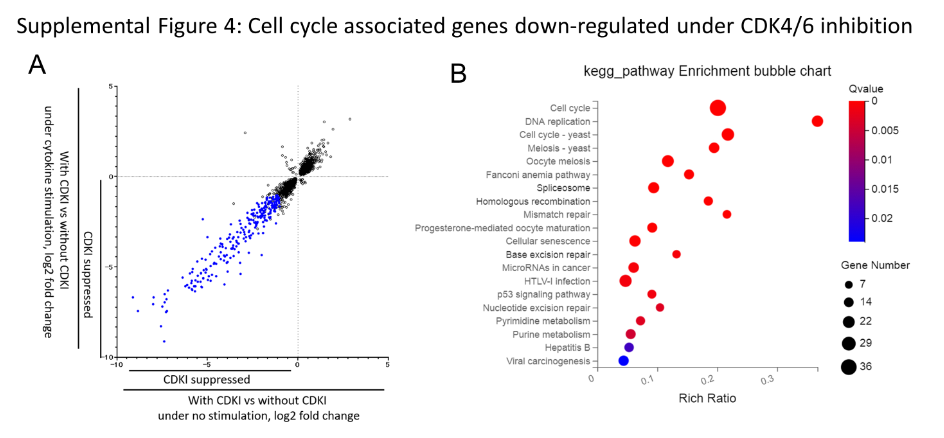


Supplementary Figure S4: Cell cycle associated genes down-regulated under CDK4/6 inhibition.

A: Scatter plot of DEGs affected by CDK4/6 inhibition with or without cytokine stimulation. Expression changes are plotted as log2 FC. We highlighted 470 genes that were repressed by both CDK4/6 with or without cytokine stimulation (Log2 FC below 0).

B: Pathway enrichment analysis of 270 genes. Color in each plot indicates the value of FDR. The number of genes included in each pathway are represented by size of plot.


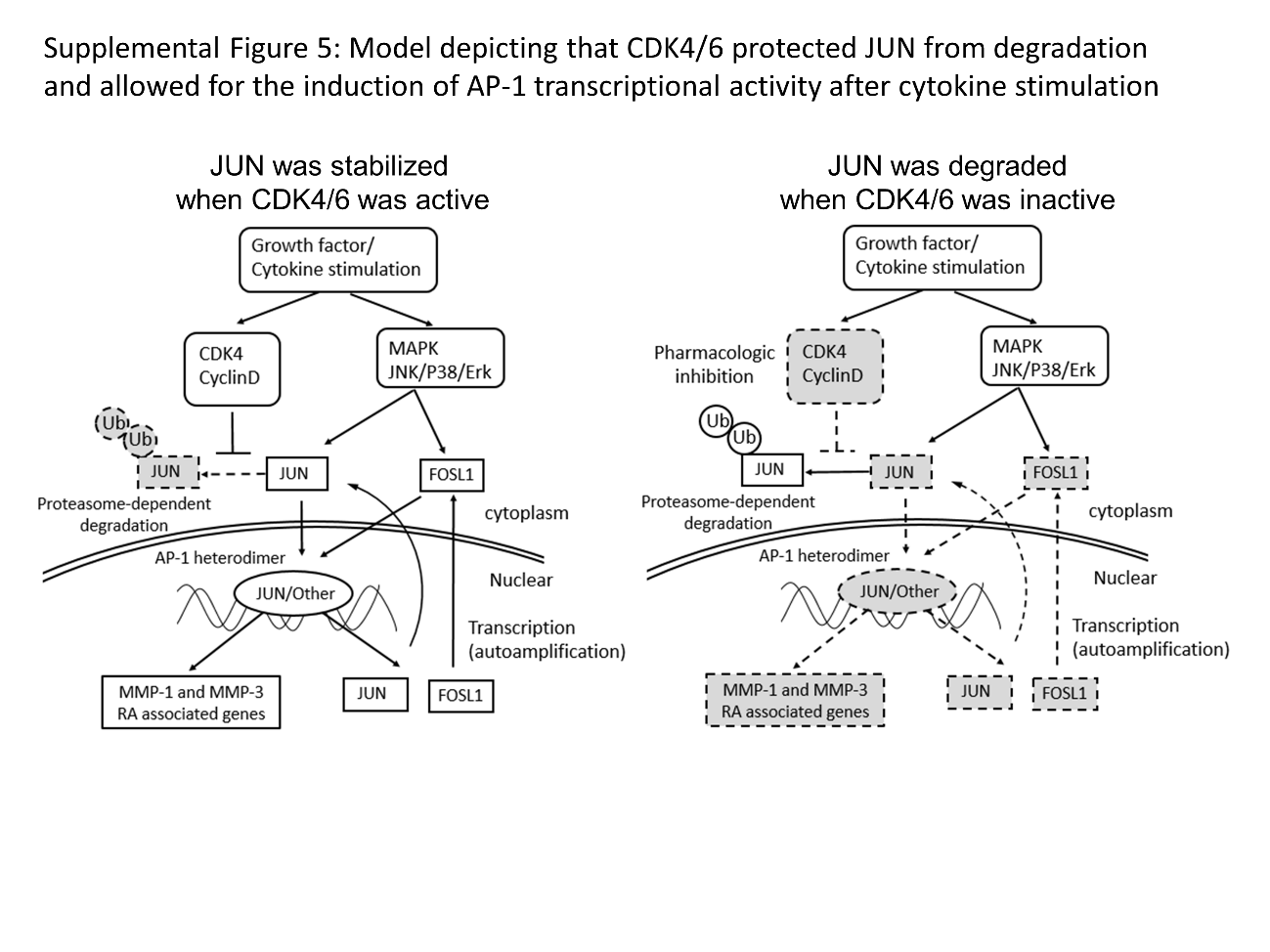


Supplementary Figure S5: Model depicting that CDK4/6 protected JUN from degradation and allowed for the induction of AP-1 transcriptional activity after cytokine stimulation.
